# Caloric Vestibular Stimulation as a Treatment for Conversion Disorder: A Case Report and Medical Hypothesis

**DOI:** 10.3389/fpsyt.2014.00063

**Published:** 2014-06-02

**Authors:** Michael Noll-Hussong, Sabrina Holzapfel, Dan Pokorny, Simone Herberger

**Affiliations:** ^1^Klinik und Poliklinik fuer Psychosomatische Medizin und Psychotherapie des Universitaetsklinikums Ulm, Ulm, Germany; ^2^Hals-Nasen-Ohrenklinik und Poliklinik, Klinikum rechts der Isar, Technische Universitaet Muenchen, Muenchen, Germany; ^3^Klinik fuer Psychosomatische Medizin und Psychotherapie des Klinikums Muenchen-Harlaching, Muenchen, Germany

**Keywords:** conversion disorder, vestibular stimulation, case report, medical hypothesis

## Abstract

Conversion disorder is a medical condition in which a person has paralysis, blindness, or other neurological symptoms that cannot be clearly explained physiologically. To date, there is neither specific nor conclusive treatment. In this paper, we draw together a number of disparate pieces of knowledge to propose a novel intervention to provide transient alleviation for this condition. As caloric vestibular stimulation has been demonstrated to modulate a variety of cognitive functions associated with brain activations, especially in the temporal–parietal cortex, anterior cingulate cortex, and insular cortex, there is evidence to assume an effect in specific mental disorders. Therefore, we go on to hypothesize that lateralized cold vestibular caloric stimulation will be effective in treating conversion disorder and we present provisional evidence from one patient that supports this conclusion. If our hypothesis is correct, this will be the first time in psychiatry and neurology that a clinically well-known mental disorder, long considered difficult to understand and to treat, is relieved by a simple or common, non-invasive medical procedure.

“[…]that mysterious leap from the soul to the body.”

Sigmund Freud, Vorlesungen zur Einführung in die Psychoanalyse, 1916–1917

… and about 100 years later:

“Perhaps the most chastening lesson […] is the striking lack of specificity of any of the findings so far described. Although we might predict that a patient describing bodily disturbance of “some kind” will exhibit abnormalities of certain candidate brain regions, we would have great difficulty modifying their diagnosis or treatment on the basis of the results of brain scanning.”

Sean A. Spence, 2006 (1)

## Introduction

Conversion disorder (2) as defined in DSM-IV (3) describes symptoms such as weakness, paralysis, seizures, or abnormal movements that are not attributable to a general medical condition or to feigning, and that are judged to be associated with psychological factors (4). It has been estimated that 10% of all mental disorders and 30% of neurological inpatients suffer from conversion disorder (2, 5, 6). However, as diagnosis is not easy (7), there are few evidence-based treatments for conversion disorder (8, 9). The name “conversion disorder” refers both to a hypothesis based on psychoanalytic etiology and to a long history of a multitude of disorders formerly named “hysteria” (10–12). Follow-up data of patients, especially those with psychogenic movement disorders revealed a persistence in abnormal movements in more than 90% of subjects. Prevalence rates of mental illness in excess of those found in the general population and in neurological disorders, plus an inability to acknowledge the essentially psychological nature of their condition characterize the outcome picture and lead to a poor longer-term prognosis in these patients (13). In special cases, the clinical phenomenology resembles the “alien hand sign” first described by Brion and Jedynak as a “feeling of estrangement between the patient and one of his hands” (14). This may, on the one hand, indicate the lack of awareness of the origin of their symptoms in conversion disorder patients as a possible result from suppression of brain activity normally related to self-agency (15), and, on the other hand, the frequent clinical impression that the symptoms are often accompanied by an affective state traditionally named “*belle indifférence*” – a condition in which the person is unconcerned with symptoms (16).

Although long dominant, the conversion hypothesis is now just one of many competing etiological hypotheses and has little supportive empirical evidence (17). Furthermore, the name “conversion disorder” has not been widely accepted by either non-psychiatrists or patients (18–20). Low education, presence of a personality disorder, and high Hamilton depression score were found to be significantly associated with conversion disorder (21). As there are many similarities between the symptoms of conversion, hysteria, and phenomena produced in hypnotic contexts, a model has been proposed that develops the idea of a central executive structure, similar to the notion of a supervisory attentional system, acting outside self-awareness but at a late stage of information processing, which can be directly influenced by both internal and external sources to produce the relevant phenomena (22). Thus, as conversion disorder, pain disorder, and dissociation disorders appear to be linked by a common mechanism, they could be classified together under the heading of “auto-suggestive disorder” (22). Altogether, the notion that the etiology of these symptoms is wholly psychological may be scientifically incorrect. For example, functional brain imaging studies that demonstrated contralateral thalamic hypoactivity in hemisensory conversion encourage us to understand conversion symptoms from a non-dualistic brain as well as mind perspective (23–27).

Caloric vestibular stimulation (CVS) is a physiological technique demonstrated to modulate tactile perception in healthy subjects after left ear CVS (28). Moreover, it has been shown that CVS is helpful, amongst others, in some patients with central pain like Dejerine–Roussy syndrome (thalamic pain syndrome) (29, 30), central post-stroke pain (31), aphasia (32), and spatial neglect (33), right-sided central pain following transverse myelitis of the cervical spinal cord (34), and post-stroke tactile allodynia (35). On the other hand, it has been shown that CVS has no effect on tinnitus (36).

Interestingly, CVS could also transiently improve hemianesthesia in right brain-damaged patients (37–39). Recent studies suggest that these effects are based on the anatomical overlap between vestibular and tactile projections (37) in the human brain. For example, in a right brain-damaged patient with neglect, this symptom was improved especially following left CVS, and worsened following right CVS. No modification of neglect was observed after bilateral vestibular stimulation (40).

The first case of transitory attenuation of neglect phenomena during CVS was documented by Silberpfennig (41) in 1941. The finding has been confirmed by Rubens by testing unilateral neglect on tasks requiring visual exploration of extrapersonal space in a series of patients (42). Cappa et al. demonstrated the positive effects of vestibular stimulation on personal and extrapersonal neglect, respectively, and on awareness of disease in four patients with severe neglect and anosognosia, and speculated about a possible role of vestibular stimulation in hemispheric brain activation (43). The effects of vestibular stimulation on somatophrenic delusion were investigated by Bisiach et al. in a patient suffering from fronto-temporo-parietal infarction located in the right hemisphere (44). They could show that transitory remission of the patient’s delusional belief was consistently observed during unilateral vestibular activation, achieved by cold-water irrigation of the contralesional ear (43). Ramachandran replicated these results in one patient with anosognosia in parietal lobe syndrome (45), and recently – as there may be a unitary mechanism underlying both anosognosia (46) and unrealistic optimism – it has been shown that left-sided cold-water CVS attenuates unrealistic optimism in healthy adults (47).

Altogether, and focusing on mental disorders in particular, it has recently been shown that a single-session CVS may have short-lived beneficial effects in mania and perhaps in other types of psychoses (48). Recently, the beneficial effects of CVS on denial of illness and manic delusions in schizoaffective disorder have been shown in a case series of three patients (48). Body integrity image disorder (BIID) is characterized by a feeling of mismatch between the internal feeling of how one’s body should be and the physical reality of how it actually is. Patients with this condition have an often-overwhelming desire for amputation of a specific limb at a specific level. Such patients are not psychotic or delusional; however, they do express an inexplicable emotional abhorrence to the limb they wish removed. It is also known that such patients show a left-sided preponderance for their desired amputation – conversion disorders are also prevalent on the left side, especially in women (49). Often, BIID patients take drastic action to be rid of the offending limb. Given the left-sided bias, emotional rejection, and specificity of desired amputation, we suggest that there are clear similarities to be drawn between BIID and somatoparaphrenia. In this rare condition, which follows a right parietal stroke, the patient rejects (usually) his left arm as “alien” (50). Ramachandran et al. go on to hypothesize that a dysfunction of the right parietal lobe is also the cause of BIID and suggest that this leads to an uncoupling of the construct of one’s body image in the right parietal lobe from how one’s body physically is (50, 51). Recently, and interestingly in healthy subjects, it has been shown that CVS, depending on the side of stimulation, has a modulating effect on mood and affective control (52).

Taking all the disparate pieces of the mentioned knowledge together, there is reason to speculate about the positive effects of CVS in special mental disorders like conversion disorder, somatoform (pain) disorders, dissociative disorder, and even posttraumatic stress disorder (PTSD) in terms of a “functional neglect” that shares a lot of the above-mentioned symptoms like unclear functional losses, perturbed affective states, and emotional peculiarities. In this pilot study, we tested for the first time, in one patient, the idea that single-session CVS by application of cold-water to the left ear induces a clinically significant, short-lived beneficial effect on conversion disorder.

## Case Report

We have provisional data from one 26-year-old right-handed male patient with conversion disorder or psychogenic movement disorder diagnosed using Fahn and Williams’ criteria (53) in terms of a hyperkinetic disturbance of voluntary motor function (54), especially of his arms and, varying, the upper part of the body (e.g., involuntary “bowings”), with more than 2 years’ duration. The German patient had received many different medical treatments with no longer lasting improvement, resulting in a significant burden for the patient and his family. In some cases, physical activity alleviated the symptoms, as has been described elsewhere (55), as (interpersonal) “stress” could trigger the symptoms.

### Behavioral instruments

Patient burden was controlled at the beginning using “The Symptom Checklist-90-R (SCL-90-R)” (56, 57), the scales for emotion experience (SEE), emotional competence questionnaire (EKF), Beck’s depression inventory (BDI), and temperament and character inventory (TCI). According to the homepage of the publishing house Pearson Assessments (58), “the symptom checklist-90-R (SCL-90-R) instrument helps evaluate a broad range of psychological problems and symptoms of psychopathology. The instrument is also useful in measuring patient progress or treatment outcomes.” The 90 items of the self-assessment checklist are scaled from 0 to 4 and is associated with the problems the patient has been suffering during the last 7 days. The summarizing global severity index (GSI) is a *de facto* standard for psychotherapy clinical practice and research, and serves as a kind of “symptom severity thermometer.” The nine specific subscales of the GSI provide an overview of the spectrum of patient complaints.

The SEE (59) assesses individual emotional experiences as well as appraisal and regulation of emotions on a five-point Likert scale including “1 = not true at all,” “2 = hardly true,” “3 = moderate true,” “4 = fairly true,” and “5 = absolute true.” The questionnaire comprises 42 items, which constitute seven subscales: (1) acceptance of emotions assesses a positive appreciation of one’s own feelings; (2) experience of emotion flooding describes the experience of too many concurrent feelings; (3) experience of emotion deficit contains items describing individuals who perceive few emotions and feel cut off from their body; (4) body-related symbolization of emotions refers to bodily sensations that denote mental processes and feelings; (5) imaginative symbolization of emotions contains items that see fantasies and dreams as helpful for coping with diverse problems; (6) experience of emotion regulation refers to the ability to regulate emotions and moods by calming oneself or getting into a lively mood; and (7) experience of self-control assesses the ability to appear outwardly controlled and self-possessed (60).

The EKF (61) comprises 62 items and participants rate on a five-point Likert-type scale the extent to which they agree (ranging from strongly agree to strongly disagree) with the statements depicted. The questionnaire consists of four subscales measuring the constructs recognizing and understanding one’s own emotions, recognizing and understanding the emotions of others, the ability to regulate and control one’s own emotions, and emotional expressiveness. The total score reflects a person’s self-rated emotional competence as a subdomain of their self-concept (62).

The applied BDI-II is a 21-item self-report instrument that measures cognitive and endogenous aspects of depression on four-point scales ranging from 0 to 3. The standard cut-offs are as follows: 0–9 indicates no depression; 10–18 a mild depression; 19–29 a moderate depression; and >29 a severe depression. This questionnaire has undergone extensive reliability and validation studies (63, 64).

The German version (65) of the TCI consists of seven factors: four dimensions of temperament (novelty seeking, harm avoidance, reward dependence, and persistence) and three dimensions of character (self-directedness, cooperativeness, and self-transcendence). It is a self-assessment tool with 240 true/false items (66). The temperament subdimensions can be divided into the following parts: (1) novelty seeking NS (subscales: exploratory excitability, impulsiveness, extravagance, and disorderliness); (2) harm avoidance HS (anticipatory worry, fear of uncertainty, shyness, and fatigability); (3) reward dependence RD (sentimentality, attachment, and dependence); and (4) persistence (P).

The character subdimensions can be divided into the following parts: (5) self-directedness SD (responsibility, purpose, resourcefulness, self-acceptance, and enlightened second nature); (6) cooperativeness C (social acceptance, empathy, helpfulness, compassion, and pure-hearted conscience); and (7) self-transcendence ST (self-forgetfulness, transpersonal identification, and spiritual acceptance).

**Figure 1:**
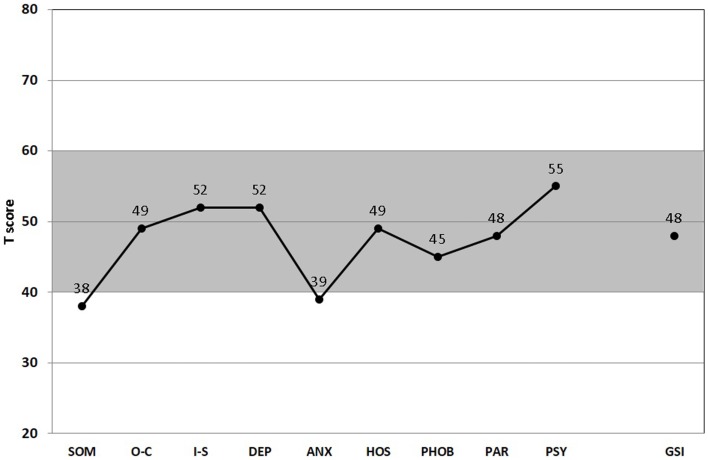
**Score *Profile* of the symptom checklist-90-R (SCL-90-R)**. All nine subscales (SOM, somatization; O–C, obsessive–compulsive; I-S, interpersonal sensitivity; DEP, depression; ANS, anxiety; HOS, hostility; PHOB, phobic anxiety; PAR, paranoid ideation; PSY, psychoticism), and the GSI (global severity index) are presented (67). (T50, T60, T70: thresholds for the standard male German population. T60 is considered “clinically suspicious” and T70 “clinically relevant.” For the GSI, T60 is already considered “clinically relevant.” Gray-shaded area depicts the normal range.)

**Figure 2:**
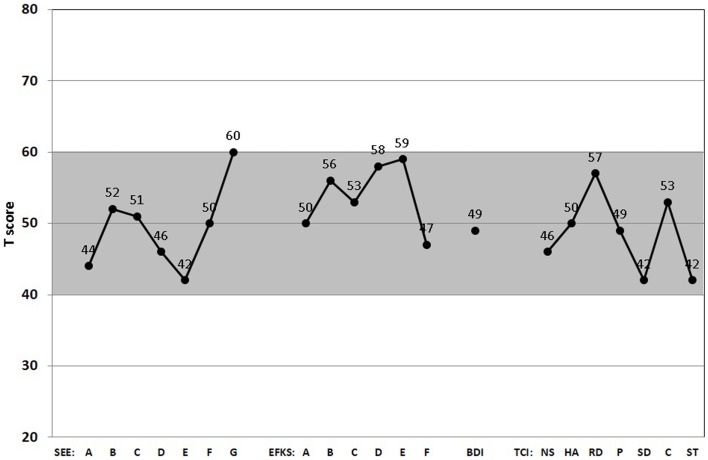
**Score *Profiles* of the scales for emotion experience (SEE), emotional competence questionnaire (EKF), Beck’s depression inventory (BDI), and temperament and character inventory (TCI)**. All relevant subscales of the SEE (A, acceptance of one’s emotions; B, experience of emotion flooding; C, experience of emotion deficiency; D, body-related symbolization of emotions; E, imaginative symbolization of emotions; F, experience of emotion regulation; G, experience of self-control), EKF (A, recognizing and understanding one’s emotions; B, recognizing emotions in others; C, regulation and control of one’s emotions; D, emotional expressivity; E, regulation of the feelings of others; F, adjustment to feelings), BDI-II, and TCI (NS, novelty seeking; HS, harm avoidance; RD, reward dependence; P, persistence; SD, self-directedness; C, cooperativeness; ST, self-transcendence) are presented. (Gray-shaded area depicts the normal range.)

The gray-shaded area between standardized *T*-scores 40 and 60 shown in Figures 1 and 2 corresponds roughly to the middle 68.3% proportion of the German population. Interestingly, all of the psychometric results of the patient were almost in this “normal” range. Being compared with the population, the scale values of the patient were neither too high, nor too low. In fact, it happens rather rarely that all results of relevant subscales lay in the “main stream field.” Hence, from the psychometric point of view (despite the clinical and subjective impression of the sufferer), the patient seems to be “suspiciously healthy.” Last not least (and “*primum nil nocere*”), no adverse reactions were reported.

### Patient reports (excerpts, in English translation)

- First trial, 30°C water, 20 s, left CVS:

“[…]I had slight symptoms when I went to the otolaryngology department. The ladies there were very nice. The treatment itself was unproblematic. Only the left ear was exposed to “cold” water. My sense of balance was minimally disturbed when I was lying down. This was over after about 2 minutes. I was walking down the hall back to the ward again at 8:39. I of course tried to evoke symptoms by repeating “I remember…” – but to no avail. No symptoms appeared when I attempted to consciously evoke them. I just couldn’t really do it. That is, I was able to think the thought through. I’ll try explaining it better next week. I didn’t want to get too euphoric, though. And unfortunately, rightly so. Today, shortly after 8 p.m., I was back at square one. I had just finished showering and was once again trying my “I remember…,” and since then, the usual “rules of the game” from before the otolaryngological checkup have applied. I’ll describe this situation in words better next week as well.”

- Second trial, 1 week later, 20°C water, 30 s, left CVS:

“I went to the otolaryngology department today. There were some technical problems at first [that lasted for an hour], then the stimulation was conducted. The desired results were unfortunately not achieved. The symptoms could not be interrupted. I can of course only speculate why it didn’t work out this time. I was “stressed” about an appointment I had at 9:00, which I would be late to [because of the technical problems mentioned above]. A symptom-triggering situation unintentionally crossed my mind during the stimulation. The interruption last time was a coincidence. Which I find difficult to imagine, however.”

- Third and final trial, 1 week later, 20°C water, 60 s, left CVS:

“My expectations today were very limited. […] I have virtually no symptoms at the moment, i.e., I am not reacting very strongly. I definitely do, however, have enough symptoms to be able to assess the effects of a stimulation. Now about the stimulation: it again had the effect that I could talk about the “handshaking situation” or similar situations or to consciously evoke them without developing any symptoms. “Stress” and “minor allegations” don’t seem to be causing me any problems so far. I just took a chance and arrived nearly 10 minutes late to my relational care appointment. Whether this would have also been possible without the stimulation is not determinable. I had a slight relapse, however, at 13:44, which was prompted by the relational counseling session. A terse allegation, which has nothing to do with the above, but it occurred when the relational counseling session came to an end. I wished my counselor a nice vacation, even though I’ll see her again next week for the nightshift. So, too soon (minor allegation). It all came back to me again when I was in my room at 13:44 and triggered slight symptoms. That’s when I thought to myself “there goes the stimulation’s effect,” since I was briefly confronted with recollections again from that point on, a bit stronger than before the stimulation. But I was already used to it from the first stimulation, when the “effect” began to diminish after 36 h. I wanted to write you a mail thereafter. But after 2 min, I was no longer responding to recollections of movements and therefore waited until now. I have not had any further movement disorders so far. Not even when the content of this mail briefly disappeared;). I am curious how long it’ll last. I don’t plan to force the issue, though.”

3 days later:

“I have not noticed any further real movement disorders so far. There were only some very slight indications, expressed more internally through minimal tension or a very slowed and inhibited movement disorder which only manifests itself very rarely, i.e., 2–3 times since Friday. I am less prone to “stress,” i.e., stressful situations are no longer accompanied by movement disorders, which allows you to act in a completely different mode.”

## Discussion

In our patient, left caloric irrigation caused an immediate and sustained relief from his functional disability that has so far lasted at least for hours. It has been stated that the vestibular system is unique among the senses because of the entirely multisensory nature of its cortical projections (68) and its overlapping with other neural networks, e.g., emotional circuits (69). In this context, it has recently been hypothesized that the human vestibular cortex may be crucial for bodily self-consciousness (70).

Neuroanatomical and neuroimaging studies have shown that vestibular stimulation activates somatosensory areas, and particularly the so-called parieto-insular vestibular cortex in the monkey, while deactivating visual areas (68). Further, recent psychophysical studies have shown that vestibular stimulation facilitates detection of electrocutaneous stimuli, suggesting a vestibular–somatosensory perceptual interaction (68, 71). While several recent reports have described the capacity of CVS to transiently improve or reverse a wide range of attentional, cognitive, and motor impairments, e.g., in mentally retarded, emotionally disturbed, and learning-disabled individuals (72) or autistic children (73), most examples are in right hemisphere-damaged patients with long-standing brain injury (37–39). Typically, patients have been tested several months or years after the onset of the deficit. A possible mechanism for the temporary reintegration of multiple cognitive functions is that vestibular stimulation activates thalamocortical mechanisms that reintegrate impaired cortical regions (74). In this context, it has been hypothesized that the essential aspect leading to neglect in brain-damaged patients is a disturbance of those cortical structures that are crucial for transforming the sensory input coordinates from the peripheral sensory organs into an egocentric, body-centered coordinate frame of reference (75), supporting a “representational theory of neglect” (76, 77). Furthermore, it has been shown that caloric stimulation impairs performance in a high-resolution mental imagery and mental rotation task, respectively, but not another cognitive task (78), and one may consequently speculate about the special power of mental imagery – and derived psychotherapeutic procedures – in some mental disorders. Last but not least, an *in vivo* microdialysis study has shown that vestibular stimulation with ice water increases acetylcholine release from rat hippocampus to 160% compared with the basal rate (79), which could reflect one facet of memory facilitation in the mentioned mental disorders (like conversion disorder, but perhaps also PTSD). However and as a limitation – but in all fairness, not unique for CVS – one has to consider placebo effects (80–83).

Finally, at least two related, also unspecific neural stimulation techniques and their published benefits should be mentioned. First, as the experiments of Alessandro Volta were amongst the first to indicate that visuo-spatial function can be altered by stimulating the vestibular nerves with galvanic current, it has recently been shown that Galvanic vestibular stimulation speeds visual memory recall (84). Second, transcranial magnetic stimulation (TMS) is a method of non-invasive brain stimulation that affects the cerebral cortex but not deep structures (85). In patients with movement disorders, the most common application of TMS has been to test the excitability of connections within and among motor areas of the cortex, which has provided useful information on pathophysiology; however, inter-individual variability in the responses has resulted in difficulties in translating this method into a clinically applicable diagnostic use (85). Repeated stimulation (e.g., 1 Hz for 20 min) can result in long-term plastic changes in the motor system, which has led to increased interest in possible therapeutic applications (85). As there is no agreement on the most effective therapy for motor conversion symptoms, functional electric stimulation has also been successfully used to reverse conversion paralysis (86–89). Moreover, it has been speculated that a decrease of motor excitability during motor imagery is the electrophysiological correlate of a disturbed voluntary control in motor conversion disorder (90, 91). Remarkably, these results further indicate that this abnormality is not restricted to the clinically affected body part. One may speculate that, amongst others, vestibular stimulation (92), TMS, and even the central effects of acupuncture (93) share some features that could reintegrate the neural circuits (94) underlying affective or movement initiation and control.

## Conclusion

If our hypothesis is shown to be correct, this will be the first time in neurology and psychiatry that a potentially chronic mental disorder, long considered difficult to treat, can be transiently relieved by a simple non-invasive, conventional medical procedure. It would offer at least a new therapeutic approach to pseudoneurological syndromes and insights in their etiology. It would also, again, demonstrate the astonishing and rapid plasticity of the brain, especially in relation to psychomotor phenomena and unconscious integration of body schema (95, 96). Altogether, psychogenic movement disorders represent a unique opportunity for medical and scientific specialties to collaborate in the care of a potentially curable but significantly disabling illness (97).

## Conflict of Interest Statement

The authors declare that the research was conducted in the absence of any commercial or financial relationships that could be construed as a potential conflict of interest.

## References

[1] Spence SA All in the mind? The neural correlates of unexplained physical symptoms. Adv Psychiatr Treat (2006) 12(5):349–58 10.1192/apt.12.5.349

[2] Noll-Hussong M Henningsen P Zur Neuro-Psychosomatik der Konversion. Schweiz Arch Neurol Psychiatr (2009) 160:356–61 10.1111/j.1439-0388.1993.tb00716.x 21395703

[3] American Psychiatric Association. Diagnostic and Statistic Manual of Mental Disorders (Text Revision). 4th ed Washington, DC: American Psychiatric Publishing, Inc (2000).

[4] Stone J LaFrance WC Jr Levenson JL Sharpe M Issues for DSM-5: conversion disorder. Am J Psychiatry (2010) 167(6):626–7 10.1176/appi.ajp.2010.09101440 20516161

[5] Carson AJ Best S Postma K Stone J Warlow C Sharpe M The outcome of neurology outpatients with medically unexplained symptoms: a prospective cohort study. J Neurol Neurosurg Psychiatry (2003) 74(7):897–900 10.1136/jnnp.74.7.897 12810775 PMC1738573

[6] Nimnuan C Hotopf M Wessely S Medically unexplained symptoms: an epidemiological study in seven specialities. J Psychosom Res (2001) 51(1):361–7 10.1016/S0022-3999(01)00223-9 11448704

[7] Morgante F Edwards MJ Espay AJ Fasano A Mir P Martino D Diagnostic agreement in patients with psychogenic movement disorders. Mov Disord (2012) 27(4):548–52 10.1002/mds.24903 22488862 PMC3675653

[8] Stone J Carson A Sharpe M Functional symptoms in neurology: management. J Neurol Neurosurg Psychiatry (2005) 76(Suppl 1):i13–21 10.1136/jnnp.2004.061655 15718216 PMC1765682

[9] Ruddy R House A Psychosocial interventions for conversion disorder. Cochrane Database Syst Rev (2005) (4):CD005331 16235402 10.1002/14651858.CD005331.pub2

[10] Bogousslavsky J Hysteria after Charcot: back to the future. Front Neurol Neurosci (2011) 29:137–61 10.1159/000321783 20938153

[11] Mai FM “Hysteria” in clinical neurology. Can J Neurol Sci (1995) 22(2):101–10 7627910 10.1017/s0317167100040166

[12] Gould R Miller BL Goldberg MA Benson DF The validity of hysterical signs and symptoms. J Nerv Ment Dis (1986) 174(10):593–7 10.1097/00005053-198610000-00003 3760849

[13] Feinstein A Stergiopoulos V Fine J Lang AE Psychiatric outcome in patients with a psychogenic movement disorder: a prospective study. Neuropsychiatry Neuropsychol Behav Neurol (2001) 14(3):169–76 11513100

[14] Goldberg G Bloom KK The alien hand sign. Localization, lateralization and recovery. Am J Phys Med Rehabil (1990) 69(5):228–38 10.1097/00002060-199010000-00002 2222983

[15] Blakemore RL Hyland BI Hammond-Tooke GD Anson JG Distinct modulation of event-related potentials during motor preparation in patients with motor conversion disorder. PLoS One (2013) 8(4):e62539 10.1371/journal.pone.0062539 23626829 PMC3633887

[16] Stone J Smyth R Carson A Warlow C Sharpe M La belle indifference in conversion symptoms and hysteria: systematic review. Br J Psychiatry (2006) 188:204–9 10.1192/bjp.188.3.204 16507959

[17] Nicholson TR Stone J Kanaan RA Conversion disorder: a problematic diagnosis. J Neurol Neurosurg Psychiatry (2011) 82(11):1267–73 10.1136/jnnp.2008.171306 21036784

[18] Espay AJ Goldenhar LM Voon V Schrag A Burton N Lang AE Opinions and clinical practices related to diagnosing and managing patients with psychogenic movement disorders: an international survey of movement disorder society members. Mov Disord (2009) 24(9):1366–74 10.1002/mds.22618 19425106

[19] Friedman JH LaFrance WC Jr Psychogenic disorders: the need to speak plainly. Arch Neurol (2010) 67(6):753–5 10.1001/archneurol.2010.91 20558396

[20] Kanaan R Armstrong D Barnes P Wessely S In the psychiatrist’s chair: how neurologists understand conversion disorder. Brain (2009) 132(Pt 10):2889–96 10.1093/brain/awp060 19321463 PMC2759333

[21] Binzer M Andersen PM Kullgren G Clinical characteristics of patients with motor disability due to conversion disorder: a prospective control group study. J Neurol Neurosurg Psychiatry (1997) 63(1):83–8 10.1136/jnnp.63.1.83 9221972 PMC2169635

[22] Oakley DA Hypnosis and conversion hysteria: a unifying model. Cogn Neuropsychiatry (1999) 4(3):243–65 10.1080/135468099395954 16571508

[23] Vuilleumier P Chicherio C Assal F Schwartz S Slosman D Landis T Functional neuroanatomical correlates of hysterical sensorimotor loss. Brain (2001) 124(Pt 6):1077–90 10.1093/brain/124.6.1077 11353724

[24] Ellenstein A Kranick SM Hallett M An update on psychogenic movement disorders. Curr Neurol Neurosci Rep (2011) 11(4):396–403 10.1007/s11910-011-0205-z 21559795 PMC4747629

[25] Lang AE Voon V Psychogenic movement disorders: past developments, current status, and future directions. Mov Disord (2011) 26(6):1175–86 10.1002/mds.23571 21626561

[26] Edwards MJ Bhatia KP Functional (psychogenic) movement disorders: merging mind and brain. Lancet Neurol (2012) 11(3):250–60 10.1016/S1474-4422(11)70310-6 22341033

[27] Nowak DA Fink GR Psychogenic movement disorders: aetiology, phenomenology, neuroanatomical correlates and therapeutic approaches. Neuroimage (2009) 47(3):1015–25 10.1016/j.neuroimage.2009.04.082 19426818

[28] Ferre ER Sedda A Gandola M Bottini G How the vestibular system modulates tactile perception in normal subjects: a behavioural and physiological study. Exp Brain Res (2011) 208(1):29–38 10.1007/s00221-010-2450-9 20972670

[29] Ramachandran VS McGeoch PD Williams L Arcilla G Rapid relief of thalamic pain syndrome induced by vestibular caloric stimulation. Neurocase (2007) 13(3):185–8 10.1080/13554790701450446 17786778

[30] Ramachandran VS McGeoch PD Williams L Can vestibular caloric stimulation be used to treat Dejerine-Roussy syndrome? Med Hypotheses (2007) 69(3):486–8 10.1016/j.mehy.2006.12.013 17321064

[31] McGeoch PD Williams LE Lee RR Ramachandran VS Behavioural evidence for vestibular stimulation as a treatment for central post-stroke pain. J Neurol Neurosurg Psychiatry (2008) 79(11):1298–301 10.1136/jnnp.2008.146738 18550629

[32] Wilkinson D Morris R Milberg W Sakel M Caloric vestibular stimulation in aphasic syndrome. Front Integr Neurosci (2013) 7:99 10.3389/fnint.2013.00099 24391559 PMC3870329

[33] Sturt R David Punt T Caloric vestibular stimulation and postural control in patients with spatial neglect following stroke. Neuropsychol Rehabil (2013) 23(2):299–316 10.1080/09602011.2012.755831 23305103

[34] McGeoch PD Ramachandran VS Vestibular stimulation can relieve central pain of spinal origin. Spinal Cord (2008) 46(11):756–7 10.1038/sc.2008.47 18521097

[35] McGeoch PD Williams LE Song T Lee RR Huang M Ramachandran VS Post-stroke tactile allodynia and its modulation by vestibular stimulation: a MEG case study. Acta Neurol Scand (2009) 119(6):404–9 10.1111/j.1600-0404.2008.01106.x 18853944

[36] Baguley DM Knight R Bradshaw L Does caloric vestibular stimulation modulate tinnitus? Neurosci Lett (2011) 492(1):52–4 10.1016/j.neulet.2011.01.052 21295114

[37] Bottini G Paulesu E Sterzi R Warburton E Wise RJ Vallar G Modulation of conscious experience by peripheral sensory stimuli. Nature (1995) 376(6543):778–81 10.1038/376778a0 7651537

[38] Bottini G Paulesu E Gandola M Loffredo S Scarpa P Sterzi R Left caloric vestibular stimulation ameliorates right hemianesthesia. Neurology (2005) 65(8):1278–83 10.1212/01.wnl.0000182398.14088.e8 16247057

[39] Bottini G Sterzi R Paulesu E Vallar G Cappa SF Erminio F Identification of the central vestibular projections in man: a positron emission tomography activation study. Exp Brain Res (1994) 99(1):164–9 10.1007/BF00241421 7925790

[40] Rode G Tilikete C Luaute J Rossetti Y Vighetto A Boisson D Bilateral vestibular stimulation does not improve visual hemineglect. Neuropsychologia (2002) 40(7):1104–6 10.1016/S0028-3932(01)00187-7 11900761

[41] Silberpfennig J Contributions to the problems of eye movement. Confin Neurol (1941) 4:1–13 10.1159/000106147

[42] Rubens AB Caloric stimulation and unilateral visual neglect. Neurology (1985) 35(7):1019–24 10.1212/WNL.35.7.1019 4010940

[43] Cappa S Sterzi R Vallar G Bisiach E Remission of hemineglect and anosognosia during vestibular stimulation. Neuropsychologia (1987) 25(5):775–82 10.1016/0028-3932(87)90115-1 3501552

[44] Bisiach E Rusconi ML Vallar G Remission of somatoparaphrenic delusion through vestibular stimulation. Neuropsychologia (1991) 29(10):1029–31 10.1016/0028-3932(91)90066-H 1762671

[45] Ramachandran VS Anosognosia in parietal lobe syndrome. Conscious Cogn (1995) 4(1):22–51 10.1006/ccog.1995.1002 7497101

[46] Ronchi R Rode G Cotton F Farne A Rossetti Y Jacquin-Courtois S Remission of anosognosia for right hemiplegia and neglect after caloric vestibular stimulation. Restor Neurol Neurosci (2013) 31(1):19–24 10.3233/RNN-120236 23142813

[47] McKay R Tamagni C Palla A Krummenacher P Hegemann SC Straumann D Vestibular stimulation attenuates unrealistic optimism. Cortex (2013) 49(8):2272–5 10.1016/j.cortex.2013.04.005 23725596

[48] Levine J Toder D Geller V Kraus M Gauchman T Puterman M Beneficial effects of caloric vestibular stimulation on denial of illness and manic delusions in schizoaffective disorder: a case report. Brain Stimul (2011) 5(3):267–73 10.1016/j.brs.2011.03.004 21783454

[49] Smokler IA Shevrin H Cerebral lateralization and personality style. Arch Gen Psychiatry (1979) 36(9):949–54 10.1001/archpsyc.1979.01780090035004 464744

[50] Ramachandran VS McGeoch P Can vestibular caloric stimulation be used to treat apotemnophilia? Med Hypotheses (2007) 69(2):250–2 10.1016/j.mehy.2006.12.013 17292561

[51] McGeoch PD Brang D Song T Lee RR Huang M Ramachandran VS Xenomelia: a new right parietal lobe syndrome. J Neurol Neurosurg Psychiatry (2011) 82(12):1314–9 10.1136/jnnp-2011-300224 21693632

[52] Preuss N Hasler G Mast FW Caloric vestibular stimulation modulates affective control and mood. Brain Stimul (2014) 7(1):133–40 10.1016/j.brs.2013.09.003 24139868

[53] Fahn S Williams DT Psychogenic dystonia. Adv Neurol (1988) 50:431–55 3400501

[54] Reich SG Pearls: hyperkinetic movement disorders. Semin Neurol (2010) 30(1):15–22 10.1055/s-0029-1245005 20127576

[55] Dallocchio C Arbasino C Klersy C Marchioni E The effects of physical activity on psychogenic movement disorders. Mov Disord (2010) 25(4):421–5 10.1002/mds.22952 20108357

[56] Derogatis LR Cleary PA Confirmation of the dimensional structure of the SCL-90: a study in construct validation. J Clin Psychol (1977) 33:981–9 10.1002/1097-4679(197710)33:4<981::AID-JCLP2270330412>3.0.CO;2-0

[57] Marcelissen TA Leong RK Nieman FH van Lankveld JJ van Kerrebroeck PE de Wachter SG Psychological and psychiatric factors as predictors for success in sacral neuromodulation treatment. BJU Int (2011) 108(11):1834–8 10.1111/j.1464-410X.2011.10205.x 21810157

[58] Available from: http://psychcorp.pearsonassessments.com/HAIWEB/Cultures/en-us/Productdetail.htm?Pid=PAg514

[59] Behr M Becker M SEE – Skalen zum Erleben von Emotionen (Scales for Emotion Experience). Göttingen: Hogrefe (2004).

[60] Werner NS Duschek S Schandry R Relationships between affective states and decision-making. Int J Psychophysiol (2009) 74(3):259–65 10.1016/j.ijpsycho.2009.09.010 19808059

[61] Rindermann H EKF – Emotionale-Kompetenz-Fragebogen – Einschätzung emotionaler Kompetenzen und emotionaler Intelligenz aus Selbst- und Fremdsicht. Göttingen: Hogrefe (2009).

[62] Lemenager T Gwodz A Richter A Reinhard I Kammerer N Sell M Self-concept deficits in massively multiplayer online role-playing games addiction. Eur Addict Res (2013) 19(5):227–34 10.1159/000345458 23428827

[63] Hautzinger M The Beck depression inventory in clinical practice. Nervenarzt (1991) 62(11):689–96 1770969

[64] Otti A Guendel H Wohlschlager A Zimmer C Noll-Hussong M Frequency shifts in the anterior default mode network and the salience network in chronic pain disorder. BMC Psychiatry (2013) 13:84 10.1186/1471-244X-13-84 23497482 PMC3616999

[65] Richter J Eisemann M Richter G Cloninger C Das Temperament- und Charakter-Inventar (TCI). Deutsche Übersetzung und Bearbeitung. Frankfurt: Swets (1999).

[66] Cloninger CR The Temperament and Character Inventory (TCI): A Guide to its Development and Use. St. Louis, MO: Center for Psychobiology of Personality, Washington University (1994).

[67] Noll-Hussong M Autenrieth M Pokorny D Herberger S Huber D The subject, its biology, and the chronic recurrent cystitis. Case Rep Psychiatry (2012) 2012:601705 10.1155/2012/601705 22934220 PMC3420663

[68] Ferre ER Bottini G Haggard P Vestibular inputs modulate somatosensory cortical processing. Brain Struct Funct (2012) 217(4):859–64 10.1007/s00429-012-0404-7 22466455

[69] Preuss N Mast FW Hasler G Purchase decision-making is modulated by vestibular stimulation. Front Behav Neurosci (2014) 8:51 10.3389/fnbeh.2014.00051 24600365 PMC3928537

[70] Lopez C A neuroscientific account of how vestibular disorders impair bodily self-consciousness. Front Integr Neurosci (2013) 7:91 10.3389/fnint.2013.00091 24367303 PMC3853866

[71] Bottini G Gandola M Sedda A Ferre ER Caloric vestibular stimulation: interaction between somatosensory system and vestibular apparatus. Front Integr Neurosci (2013) 7:66 10.3389/fnint.2013.00066 24062651 PMC3774982

[72] Weeks ZR Effects of the vestibular system on human development, part 2: effects of vestibular stimulation on mentally retarded, emotionally disturbed, and learning-disabled individuals. Am J Occup Ther (1979) 33(7):450–7 224686

[73] Freeman BJ Frankel F Ritvo ER The effects of response contingent vestibular stimulation on the behavior of autistic and retarded children. J Autism Child Schizophr (1976) 6(4):353–8 10.1007/BF01537912 1087304

[74] Schiff ND Pulver M Does vestibular stimulation activate thalamocortical mechanisms that reintegrate impaired cortical regions? Proc Biol Sci (1999) 266(1417):421–3 10.1098/rspb.1999.0654 10097398 PMC1689689

[75] Karnath HO Subjective body orientation in neglect and the interactive contribution of neck muscle proprioception and vestibular stimulation. Brain (1994) 117(Pt 5):1001–12 10.1093/brain/117.5.1001 7953584

[76] Rode G Perenin MT Temporary remission of representational hemineglect through vestibular stimulation. Neuroreport (1994) 5(8):869–72 10.1097/00001756-199404000-00004 8061285

[77] Geminiani G Bottini G Mental representation and temporary recovery from unilateral neglect after vestibular stimulation. J Neurol Neurosurg Psychiatry (1992) 55(4):332–3 10.1136/jnnp.55.4.332-a 1583527 PMC489058

[78] Mast FW Merfeld DM Kosslyn SM Visual mental imagery during caloric vestibular stimulation. Neuropsychologia (2006) 44(1):101–9 10.1016/j.neuropsychologia.2005.04.005 15896815 PMC1661665

[79] Horii A Takeda N Mochizuki T Okakura-Mochizuki K Yamamoto Y Yamatodani A Effects of vestibular stimulation on acetylcholine release from rat hippocampus: an in vivo microdialysis study. J Neurophysiol (1994) 72(2):605–11 7983522 10.1152/jn.1994.72.2.605

[80] Miller FG Colloca L Kaptchuk TJ The placebo effect: illness and interpersonal healing. Perspect Biol Med (2009) 52(4):518–39 10.1353/pbm.0.0115 19855122 PMC2814126

[81] Hrobjartsson A Gotzsche PC Placebo interventions for all clinical conditions. Cochrane Database Syst Rev (2004) (3):CD003974 10.1002/14651858.CD003974.pub2 15266510

[82] Enck P Benedetti F Schedlowski M New insights into the placebo and nocebo responses. Neuron (2008) 59(2):195–206 10.1016/j.neuron.2008.06.030 18667148

[83] Baik JS Han SW Park JH Lee MS Psychogenic paroxysmal dyskinesia: the role of placebo in the diagnosis and management. Mov Disord (2009) 24(8):1244–5 10.1002/mds.22509 19353684

[84] Wilkinson D Nicholls S Pattenden C Kilduff P Milberg W Galvanic vestibular stimulation speeds visual memory recall. Exp Brain Res (2008) 189(2):243–8 10.1007/s00221-008-1463-0 18584162

[85] Edwards MJ Talelli P Rothwell JC Clinical applications of transcranial magnetic stimulation in patients with movement disorders. Lancet Neurol (2008) 7(9):827–40 10.1016/S1474-4422(08)70190-X 18703005

[86] Chastan N Parain D Verin E Weber J Faure MA Marie JP Psychogenic aphonia: spectacular recovery after motor cortex transcranial magnetic stimulation. J Neurol Neurosurg Psychiatry (2009) 80(1):94 10.1136/jnnp.2008.154302 19091717

[87] Feinstein A Psychogenic aphonia: spectacular recovery after motor cortex transcranial magnetic stimulation. J Neurol Neurosurg Psychiatry (2009) 80(1):4 10.1136/jnnp.2008.162842 19091703

[88] Schonfeldt-Lecuona C Connemann BJ Spitzer M Herwig U Transcranial magnetic stimulation in the reversal of motor conversion disorder. Psychother Psychosom (2003) 72(5):286–8 10.1159/000071900 12920333

[89] Schonfeldt-Lecuona C Connemann BJ Viviani R Spitzer M Herwig U Transcranial magnetic stimulation in motor conversion disorder: a short case series. J Clin Neurophysiol (2006) 23(5):472–5 10.1097/01.wnp.0000219004.69158.1e 17016159

[90] Liepert J Hassa T Tuscher O Schmidt R Abnormal motor excitability in patients with psychogenic paresis. A TMS study. J Neurol (2009) 256(1):121–6 10.1007/s00415-009-0090-4 19172217

[91] Liepert J Hassa T Tuscher O Schmidt R Electrophysiological correlates of motor conversion disorder. Mov Disord (2008) 23(15):2171–6 10.1002/mds.21994 18785215

[92] Lopez C Blanke O Mast FW The human vestibular cortex revealed by coordinate-based activation likelihood estimation meta-analysis. Neuroscience (2012) 212:159–79 10.1016/j.neuroscience.2012.03.028 22516007

[93] Van Nuenen BF Wohlgemuth M Wong Chung RE Abdo WF Bloem BR Acupuncture for psychogenic movement disorders: treatment or diagnostic tool? Mov Disord (2007) 22(9):1353–5 10.1002/mds.21467 17486612

[94] Klingner CM Volk GF Flatz C Brodoehl S Dieterich M Witte OW Components of vestibular cortical function. Behav Brain Res (2013) 236(1):194–9 10.1016/j.bbr.2012.08.049 22960258

[95] Rode G Vallar G Revol P Tilikete C Jacquin-Courtois S Rossetti Y Facial macrosomatognosia and pain in a case of Wallenberg’s syndrome: selective effects of vestibular and transcutaneous stimulations. Neuropsychologia (2012) 50(2):245–53 10.1016/j.neuropsychologia.2011.11.018 22142667

[96] Lopez C Schreyer HM Preuss N Mast FW Vestibular stimulation modifies the body schema. Neuropsychologia (2012) 50(8):1830–7 10.1016/j.neuropsychologia.2012.04.008 22561888

[97] Kranick SM Gorrindo T Hallett M Psychogenic movement disorders and motor conversion: a roadmap for collaboration between neurology and psychiatry. Psychosomatics (2011) 52(2):109–16 10.1016/j.psym.2010.12.017 21397102 PMC3073765

